# Dutch citizens of Turkish origin who utilize healthcare services in Turkey: a qualitative study on motives and contextual factors

**DOI:** 10.1186/s12913-018-3026-9

**Published:** 2018-04-17

**Authors:** Aydın Şekercan, Anke J. Woudstra, Ron J. G. Peters, Majda Lamkaddem, Seval Akgün, Marie-Louise Essink-Bot

**Affiliations:** 1 Department of Public Health | Research Theme: Diversity and Quality of Care, Academic Medical Center at the University of Amsterdam, PO Box 22660, 1100 DD Amsterdam, Netherlands; 2 Department of Cardiology, Academic Medical Center at the University of Amsterdam, Amsterdam, Netherlands; 3 Department of Public Health, Başkent University Hospital at Başkent University, Ankara, Turkey

**Keywords:** Medical tourism, Cross-border care, Ethnicity, Turkish origin, Accessibility, Health services research

## Abstract

**Background:**

Dutch residents of Turkish origin frequently utilize healthcare in Turkey.

**Methods:**

To investigate their motives for doing so, we conducted a qualitative study among these healthcare users using semi-structured interviews. We complemented this with informal conversations with Turkish healthcare providers and observations at the registration offices and waiting rooms of outpatient clinics in several Turkish hospitals.

**Results:**

Respondents believed their perceived needs for referral to specialist care and diagnostic assessments to quantify their health were not being met in the Netherlands.

**Conclusions:**

These mismatches in expectations of what constitutes “good care” led to dissatisfaction with Dutch primary care. Consequently, respondents utilized healthcare in Turkey if the opportunity arose, and were encouraged in this by their social networks. Establishing cross-border communication between healthcare providers is necessary, because there is currently no continuity of care for cross-border patients.

**Electronic supplementary material:**

The online version of this article (10.1186/s12913-018-3026-9) contains supplementary material, which is available to authorized users.

## Background

Currently, 4% (19.6 million) of the total European Union (EU) population is of non-European origin [1]. In the Netherlands, 12% of the Dutch general population is of non-Western origin [2]. Previous studies have shown that European residents of non-Western origin also utilize healthcare in their country of origin [3–8]. In a previous study, we found that, compared with respondents of Moroccan, Ghanaian, and Surinamese origin, respondents of Turkish origin reported the highest healthcare utilization in the country of origin (HCUCO) [9].

Dutch citizens of Turkish origin show a more explicit orientation towards the Turkish healthcare system than other non-Western migrants in the Netherlands [9]. After adjusting for socioeconomic, cultural, language, and health status factors, the statistically significant association between being of Turkish origin and healthcare utilization in Turkey suggested specific (unidentified) factors or mechanisms underlying this group’s greater tendency for HCUCO.

Several West European countries (including Belgium, France, the Netherlands, and Germany) have relatively large Turkish migrant populations. In the 1960s and early 1970s, migration from Turkey was encouraged to fill labour shortages in unskilled occupations. A second period of migration occurred (1970–1980) during which the spouses and children of many such “guest workers” joined them in the Netherlands. The majority of the Turkish migrants came from five provinces in Turkey: Konya, Sivas, Kayseri, Nevşehir, and Ankara. Since then, many young Turkish people (second-generation migrants) have chosen ethnic concordant partners and tend to remain in contact with their culture [10]. First-generation migrants are now middle-aged or elderly. The bond with the country of origin has remained intact in a number of ways, since many of the Turkish migrants go to Turkey not only for vacations, but also for business or health purposes (i.e., they lead transnational lives). Many Turkish migrants consult medical specialists at outpatient clinics in Turkey [9].

### Turkish healthcare system

The Turkish healthcare system is characterized by a centralized governance model, with public and private hospitals providing specialist healthcare to Turkish residents. Over the past decade, to improve the overall healthcare system, Turkey has introduced changes to the system through the Health Transformation Program (HTP). The HTP focused on both the demand side (increased health insurance coverage, and expanded package coverage) and the supply side (increased health human resources, and an increase in municipal health centers and hospitals (including private hospitals)) [11]. Universal healthcare coverage (state insurance) has increased accessibility to private and state hospitals by reducing out-of-pocket costs. The HTP also introduced a stronger primary care system, in order to lessen the burden on hospitals. The HTP enables patients to choose to visit a GP, instead of going directly to a medical specialist. However, patients still have to go to the hospitals during out-of-office hours and for emergency care. For foreign patients, the healthcare coverage in private hospitals depends on the contracts between the private hospital and the insurance company in the patient’s country of residence. Currently, the only major differences between the Turkish healthcare system and the Dutch healthcare system are that the Netherlands has a referral system in which first-line practitioners provide referrals to specialist care, and a strong general practitioner (GP) system that serves as the first line of care [12, 13].

### Healthcare utilization in Turkey by Dutch citizens

In the Netherlands, all citizens have compulsory health insurance under the Health Insurance Act [14], which covers all necessary healthcare in the Netherlands and emergency healthcare abroad [15]. They are also entitled to healthcare through the Turkish government’s social insurance for foreigners from acknowledged countries (state insurance) during temporary stays if they requested an E111 form before arrival, regardless of nationality. The legal basis for the E111 form comes from the mutual agreement between the Republic of Turkey and the Kingdom of the Netherlands that has been in force since 1968 [16]. However, Dutch citizens with a Turkish nationality are automatically insured in Turkey by the universal healthcare coverage.

The E111 form is a European medical form that has been replaced by the European Health Insurance Card (EHIC) within the European Economic Area (EEA), but is still in effect in certain countries outside the EEA (e.g., Turkey, and Morocco) [17]. The EHIC or E111 form provides access to medically necessary healthcare during a temporary stay in any of the EEA countries, under the same conditions and cost as people insured in that country. Another important factor is that although the state insurance requires fewer out-of-pocket payments than the Dutch insurance, it has to be paid for in advance.

### Purpose of the study

We conducted a qualitative study in order to understand the tendency of Dutch citizens of Turkish origin to utilize healthcare in Turkey and to explore their motives for doing so. Our hypotheses for explaining the HCUCO of these people included the following: being able to bypass the referral system to specialist care in the Netherlands, previous positive experiences with the medical culture of private hospitals during visits to Turkey [4], and the perceived improvement in the quality of healthcare in Turkey acting as a pull factor for utilizing this care [18–20]. This resulted in the following research question: “Which motives and contextual factors influence the decision of Dutch citizens of Turkish origin to seek healthcare in their country of origin?”

## Methods

### Study design

We conducted semi-structured interviews with Dutch patients of Turkish origin who were utilizing healthcare in Turkey. We complemented this with informal conversations with Turkish healthcare providers, and observations at the waiting rooms and registration offices of the outpatient clinics in several Turkish hospitals. The choice for on-the-spot semi-structured interviews provided us with a unique opportunity to interview respondents before they became aware of their diagnosis and treatment, which meant that their reasons for and experiences and expectations of HCUCO had not yet been influenced by this information.

### Respondents

We interviewed 12 Dutch patients of Turkish origin in 6 hospitals in Turkey. Patients were identified as being from the Netherlands at the registration office. Before inviting a patient to join our study, we made sure the patient had a non-acute health complaint (e.g., long-standing complaints, chronic complaints) for which he/she chose to utilize healthcare in Turkey. We purposively selected patients based on age, sex, and migration generation [21]. Migration generation was classified by using country-of-birth criteria [22]. Recruitment was continued until no new themes emerged and data saturation was reached [23].

We selected six private hospitals in three large cities (two in Ankara, two in Konya, and two in Alanya), based on vacation patterns and province of origin of Dutch citizens of Turkish origin. We only selected private hospitals with known contracts with Dutch insurance companies. No State hospital in these three large cities had contracts with Dutch insurance companies, therefore Dutch patients of Turkish origin almost always prefer to go to private hospitals where their care can be reimbursed by the Dutch insurance company. Istanbul was excluded due to the fact that many Dutch patients of Turkish origin travel to their province of origin and utilize healthcare nearby.

### Data collection

After visiting the registration office and a short examination by a physician, patients had to wait for reimbursement approval from their private insurance companies for a subsequent diagnostic work-up and treatment. This took approximately one hour, during which the patient was invited to join the study. Almost all respondents tried to use their Dutch health insurance before using the Turkish state insurance, because the Turkish state insurance often requires out-of-pocket payments in advance. In the end, half of the respondents used the Dutch health insurance, in case the Dutch insurance companies perceived the care as necessary and reimbursed it. The other half used the Turkish state insurance. The first author conducted all semi-structured in-depth interviews (which lasted 30–60 min) in a private room in the hospital, between June and September 2015. The interviews were audiotaped with the respondents’ consent and transcribed verbatim.

The respondents said the first author (a bilingual Dutch resident of Turkish origin, second-generation migrant) was like one of their own children, and they were proud of him for succeeding at academic level. We believe this provided the respondents with the trust needed to discuss the topics more openly. All of the interviews were conducted in Turkish, except for one interview conducted in Dutch.

Our topic list Additional file 1 focused on the pathways to, reasons for, and expectations and consequences of HCUCO. The content of the topic list was based on the literature on HCUCO [3–8] and Andersen’s theoretical model of healthcare utilization and accessibility of care [24].

The Andersen model was developed primarily to assist in understanding why individuals utilize healthcare services [24]. The model distinguishes between system-related factors and patient-related factors, and its development has continued for over 40 years. Our study focused only on patient-related factors, which are divided into three categories: predisposing characteristics, enabling resources, and perceived need. Predisposing characteristics represent patient-related factors that increase the likelihood of using healthcare services [24]. The main factors for this category are health beliefs and knowledge about health and healthcare services; demographic factors; and social interaction. Enabling resources include factors that give a patient the opportunity to utilize care, such as having insurance and access to healthcare providers. Finally, perceived need is a patient’s assessment of his/her health status and health beliefs on when to seek care.

The topic list guiding the interviews was explorative, and based on five questions. First, we explored the respondents’ health complaints, health status, and what type of care they had utilized in the Netherlands [25]. Second, we asked respondents what motivated them to seek care in Turkey. Follow-up questions were asked to understand which motives and contextual factors had influenced their decision. We asked about system-related factors related to the Turkish healthcare system, as well as factors related to the Dutch healthcare system. Third, we asked about their expectations of Turkish healthcare. This provided us not only with more detail on their motives, but also on their beliefs about healthcare, health, and perceived need for care. Fourth, we asked respondents if they opted to choose the Dutch healthcare system for their current health complaint. This was intended to confirm their previous answers, and to be sure we had not missed any important motives. Finally, we asked the respondents about the possible consequences of cross-border care to understand whether their choice for cross-border care had been a conscious one.

Three respondents had rather low abstraction levels, which made it difficult to use open-ended questions. In these cases, closed questions were asked to confirm what was already known from earlier interviews. Situations relevant to the three respondents were described to enable them to provide their perspectives.

In addition to the interviews, the first author made observations at the hospital’s registration offices, which is the first stop for patients with non-acute complaints. This enabled him to take notes on how patients and hospital personnel interacted during the outpatient clinic visit in order to assess the healthcare pathways from patients’ perspective. Furthermore, observations were made on the hospitality and the atmosphere in the hospital. Also, after coffee breaks or at the end of staff meetings, Turkish physicians of different specialties (2 clinical, 1 surgical, 1 paediatrics) were asked, separately in their own offices, about their views on Dutch citizens of Turkish origin who utilized healthcare in their facility using the same five questions and topic list. These notes aided data analysis by providing a broader context for the patients’ responses.

### Data analysis

We analysed the transcripts and field notes from respondent interviews and informal conversations with several stakeholders (i.e., three hospital directors, one government representative, and more than ten healthcare providers) using a descriptive qualitative approach with a strong foundation in the framework method [26, 27]. Several stages can be identified in our approach: familiarization with the content of the interviews by reading and re-reading the transcripts, identifying a thematic framework, indexing, charting, and mapping the data. After the familiarization stage, the first author (AŞ) identified themes and sub-themes in respondents’ reasons, expectations, and experiences, and coded these using qualitative data analysis software (MAXQDA, Version 11, VERBI GmbH, Berlin, Germany). The relationships between the themes and sub-themes were explored, and the underlying mechanisms of HCUCO by Dutch citizens of Turkish origin were identified.

**Fig. 1:**
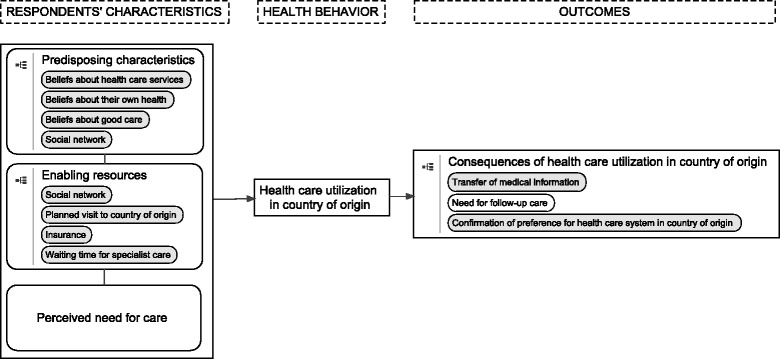
Healthcare consumption in the country of origin by residents of non-Western origin

A second qualitative researcher (AJW) simultaneously coded several interviews independently. Differences in coding and the coding tree were discussed and adjusted if both researchers (AS and AJW) agreed on the changes. If there were unresolved differences, the other authors were consulted to reach consensus; they were also asked to comment on the coding tree as a whole. To make adjustments to the model, we used the constant comparative method to examine similarities and differences between the coding tree and the Andersen model [27]. Our model became an operationalization of the Andersen model for HCUCO by citizens of non-Western origin (Fig. 1).

## Results

Half of the respondents used the Dutch health insurance and the other half used the Turkish state insurance (Table 1). Because one of the respondents (R04) was hospitalized and in poor physical condition, her husband was interviewed and the patient provided information when necessary. None of the respondents acknowledged low Dutch language proficiency as a motive for HCUCO. We used our model to describe the findings (Fig. 1).

**Table 1:** Respondents’ characteristics

Respondents	Age	Sex	Employee status	Educational level^a^	Duration of stay in Turkey	Frequency of travel to Turkey	Years of stay in the Netherlands
R01	70	M	Retired	Low	6 months	Yearly	44
R02	49	M	Employed	Low	4 weeks	Not available	35
R03	45	F	Employed	Low	Not available	Not available	30
R04	67	F	Retired	Low	11 months	Yearly	44
R05	40	M	Employed	High	3 weeks	Not available	15
R06	25	F	Homemaker	Low	7 weeks	Not available	6
R07	61	M	Employed	Low	5 weeks	Once every 5 years	42
R08	30	F	Employed	Low	Not available	Once every 2 years	Born in the Netherlands
R09	67	M	Retired	Low	6 months	Yearly	43
R10	36	M	Employed	High	2–3 weeks	4 times a year	9
R11	26	F	Employed	Low	3.5 weeks	Yearly	Born in the Netherlands
R12	45	M	Employed	High	Living in Turkey since 2004	Not applicable	12

^a^Educational level was defined as low when a respondent had no formal schooling, primary schooling only, lower vocational or lower secondary schooling, and as high when a respondent had intermediate vocational, intermediate/higher general secondary schooling or higher vocational schooling or university

### Respondents’ characteristics

#### Predisposing characteristics

##### Health beliefs

The respondents were forthcoming about their health beliefs during all of the interviews, and expressed themselves at length. These health beliefs can be divided into beliefs about healthcare services and beliefs about their own health, which lead to specific health beliefs about what constitutes good care.

##### Beliefs about healthcare services

Respondents made a clear distinction between the Turkish and the Dutch healthcare system. None of the respondents were satisfied with the referral system in the Netherlands, because they felt it impeded their access to specialist care. Respondents mentioned this referral system as a motive for seeking healthcare in Turkey, and believed that the Dutch government or health insurance companies pressured the GP to keep costs down by refusing or delaying a referral to which the patient felt entitled. Some respondents then bypassed the system by utilizing healthcare in their country of origin. For example, a patient with a weight-gain problem said the following:

*“I don’t think it was assessed properly. Only some diagnostic blood tests, they said the results were good … they’re just GPs, and it’s not easy to get referred to a hospital. That’s why more people from the Netherlands tend to go to Turkish hospitals.” (R03).*

Others utilized healthcare in Turkey in addition to their healthcare utilization in the Netherlands. They took the diagnosis to their GP in the Netherlands and asked for a referral to specialist care. Respondents who had previously utilized healthcare in Turkey said their Dutch GP always referred them to a specialist when they gave them the medical information obtained in Turkey. For example, one respondent said the following about his wife:

*“She had a herniated disk. They looked at it [in Turkey], took X-rays and 3D scans, and we took them with us. Actually, our main problem in the Netherlands was the waiting time. It can take 6 to 12 months, and in the meantime the pain is still there. So we came here, had the X-rays taken, and showed them to our GP. The GP started treatment immediately and discussed with us what he could do.” (R02).*

There was also a group who bypassed or postponed a visit to their GP in the Netherlands because they perceived the GP as being passive, and perceived the seemingly common recommendation to use acetaminophen (a standard over-the-counter painkiller) as a lack of attention. They waited for their complaints to either disappear or worsen, so that the GP would be more likely to take action (i.e., physical examination, medication other than acetaminophen, or referral) or, when it was convenient, they would wait until a planned visit to Turkey. One patient had complaints for two years and had visited her GP only twice, but because acetaminophen had been prescribed, the patient and her family decided to go to Turkey for care.

*“When they don’t give you any attention, you don’t feel like going back. You think you're going for nothing, so why go? And they always suggest taking acetaminophen. You can take up to six tablets a day.” (R06).*

Respondents had ambiguous views about the GP as the first line of contact with healthcare services. Even though they were dissatisfied at not being referred to specialist care, most respondents praised their own GP and how the first line of care was organized. The respondents saw the GP as vital, and as someone who viewed them holistically—that is, looking not only at their health problems, but also at how they were living and their well-being.

Respondents had mixed feelings about how GP care was organized. One patient appreciated the fact that it was appointment-based, which meant there was no waiting time on the day of the appointment. However, another patient complained about the fact that when she was ill, she could not see the GP immediately but had to make an appointment. One second-generation migrant said the following:

*“Making appointments, that’s really great. For example, if we make an appointment with the GP for 9:15 and we arrive at 9:10, there will be a maximum of two people ahead of you— there isn’t much waiting time. Within five minutes the GP comes out, greets you, and takes you to his office. It’s a wonderful thing.” (R08).*

The respondents’ beliefs about the Turkish healthcare system were often the opposite of their beliefs about the Dutch healthcare system. Almost all respondents reported that the way in which the healthcare system in the Netherlands was organized was clear and structured, but quite slow with regard to waiting times and referral to specialist care. However, they reported the opposite for the healthcare system in Turkey, where the respondents perceived healthcare as being organized in a way that was neither structured nor standardized. Even so, respondents praised the swift provision of services in private hospitals and direct accessibility of specialist care.

All respondents had a strong preference for private hospitals in Turkey because they perceived state hospitals as being slower at providing services and inferior in terms of quality of care. For example, with regard to the services in a private hospital, a patient with cancer of the biliary system said the following:

*“My appointment was on Thursday at 1 p.m. Around 4 or 5 p.m. everything that needed to be done was done. I slept in the hospital that night. The next day, the results slowly started coming in. On Saturday they told me there was a tumour that needed to be surgically removed because it might keep growing. The physician told me to tell my wife the news and after that we could talk again. Then they told me I could have the operation on Wednesday. So, on Wednesday I had the operation. I went to the hospital on Thursday for a check-up, and by the following Wednesday [within 6 days], I already had the operation.” (R10).*

Private hospitals have special care pathways for patients insured abroad in which appointments, diagnostics, and insurance forms can be arranged, and which allow the respondents to see the specialist on the same day. The respondents were very appreciative of the hospitality, atmosphere, and swiftness of the care provided in these hospitals.

##### Beliefs about their own health

Respondents believed that their health was an object that could be measured, altered, controlled, and expressed in numbers. We found a strong tendency among respondents to view health problems from a pathophysiological perspective rather than to look for causes outside the medical sphere or engage in preventive measures. Two respondents (R03 and R11) were not satisfied with the care they received from their GPs and their advice regarding the respondents’ unexplained weight gain, and asked for further diagnostic work-ups when standard blood analyses came back negative. They utilized healthcare in Turkey because they believed there must be a medical reason for their weight gain.

*“The GP only did some blood tests. I want to slim down. I know that won’t happen all at once. But they don’t [do anything]. They say you need to push yourself [exercise, diet].” (R03).*

*“The tests came back negative two or three times, but I told my GP I wasn’t feeling well. I told them my problems, but they told me everything was working fine. They don’t go all the way for you… They tell me the results are good, but why do I keep getting heavier?” (R11).*

When preventive measures were advised, respondents had a strong desire to quantify the effects with a diagnostic work-up. One respondent came with his wife to find out if eating more dairy products had led to higher calcium levels in their blood:

*“We had some back pain. We thought maybe it was something with our bones. Last year the Turkish healthcare provider told us there was some degeneration of the bones. They told us what to do for it, to take a vitamin pill, to eat a lot of milk, yoghurt. We did all of these things. We wanted to have another check-up to compare the results.” (R08).*

##### Beliefs about good care

Beliefs about both healthcare systems and meeting the respondents’ healthcare needs were often mentioned as “defining” good clinical practice. The Turkish healthcare system was closer to the respondents’ beliefs and healthcare needs than the Dutch system, which lead to a preference for the Turkish system. Moreover, some older respondents (who were accustomed to the Dutch healthcare system) said they perceived a decline in quality of care in the Netherlands due to healthcare budget cutbacks, which reinforced this preference. They perceived good care as being admitted to the hospital, whereas nowadays patients are more often seen on an outpatient basis, as expressed by the following respondent:

*“In the past, when something happened, they immediately admitted you to the hospital [in the Netherlands]. Now, for example, I call the GP on the nightshift and he doesn’t do anything but write a prescription for acetaminophen.” (R07).*

However, respondents were unable to give a clear definition of what type of care they perceived as being good clinical practice. A respondent whose daughter was sick changed his views on good clinical practice depending on which healthcare system met his perceived need for care. He said that a physician who “took action” on your health complaints was perceived as being a better physician. Yet, at the same time he said it would also be better to wait and see, as his Dutch GP generally told him previously:

*“In the Netherlands there is a tendency not to give medication when it isn’t needed. I like that, but, in general, Turkish people are used to going to a doctor and getting something. If you don’t get medication, an injection, or a referral to the hospital, a doctor hasn’t examined you properly.” (R05).*

Still, the same respondent perceived treatment as being necessary, even though his Dutch GP would not continue the treatment that was given in Turkey:

*“We’re giving the medication by injection. We’ve given her two already, and tomorrow will be the last one. They only gave us three syringes instead of five, because we’re going back to the Netherlands tomorrow. The doctor didn’t give us five because when you go back to the Netherlands, the doctors there don’t use this treatment. They will not continue the treatment we were getting in Turkey. So the doctor did not want to give us five syringes for nothing, but gave us three, until the day we go back. The treatment should be continued—you should have all five of them.” (R05).*

Another factor, respondents linked to good clinical practice, was the amount of time a doctor invested in a consultation or operation. A physician spending more time on a consultation, was seen as both positive and negative. Some respondents said this was better because it meant additional attention, while others saw it as an indication of lack of experience. However, none of the respondents mentioned experience with a disease or a medical specialist’s reputation as a marker of quality of care.

#### Enabling resources

The following themes emerged from the interviews as enabling resources: social network, visiting Turkey, health insurance, and waiting times in Turkey. As a predisposing characteristic, the social network influenced respondents’ motives to seek healthcare in Turkey. As an enabling resource, the social network provided assistance as informal caregivers, companions, and/or guides through the healthcare utilization process. One second-generation migrant even stated that without the help of her social network, she would not have utilized healthcare in Turkey:

*“I live at my brother’s home in the Netherlands. I couldn’t see myself coming to Turkey and doing this on my own [without his help].” (R06).*

Other enabling resources were a planned vacation to Turkey, being insured with a Dutch insurance company that had a contract with the Turkish hospital they visited, and having no waiting times for diagnostic work-ups at the Turkish outpatient clinics. It is important to emphasize that almost all respondents stated that their decision to visit a Turkish hospital was made after arriving in Turkey. Healthcare services utilization in Turkey therefore seems to be opportunistic in nature rather than deliberately planned.

#### Perceived need for care

Respondents viewed healthcare services as a commodity that should be available on demand, which can be seen as a more consumerist view of healthcare. Their beliefs on health, healthcare services, and good clinical practice led to a high perceived need for care in Turkey, since direct access to medical specialist care and fast service were perceived as unobtainable in the Netherlands. The social network reinforced their beliefs and needs. One respondent described the Dutch system as not being liberal enough and not meeting the demands of patients, who should be in charge of their own healthcare utilization:

*“Both the Netherlands and Turkey are liberal economies. This means, for example, that I have the right to access healthcare. I pay health insurance premiums. So I should have to right to choose to go directly to a medical specialist or … My choice should be respected.” (R12).*

### Health behavior

#### Healthcare utilization in Turkey

We observed that HCUCO remained limited mainly to visits to outpatient clinics, where respondents were diagnosed and prescribed medication. However, almost all respondents planned to go to their GP in the Netherlands for another second opinion. Respondents said they had a certain mistrust of Turkish medical specialists, as they had heard stories that they might also have financial incentives for treating patients. Therefore, respondents often went to their Dutch GPs to make sure the diagnosis was in line with the treatment given, as well as for a possible referral to a specialist.

### Outcomes

#### Consequences of healthcare utilization in Turkey

The respondents reported that the main consequences of healthcare utilization in Turkey were that no arrangements were made for follow-up care and that transferring medical information to the GP in the Netherlands was their own responsibility. One participant said that arranging follow-up care in the Netherlands was quite cumbersome, and he therefore avoided treatment in Turkey if it was not urgent.

*“I would have my operation in the Netherlands for one reason. The doctor in the Netherlands will be reluctant to help [when you come for a follow-up after being treated in Turkey]. The Dutch doctor will tell you, ‘You’re living in the Netherlands, so you need to be treated here. Why do you have your operation in Turkey, but then want me to do the follow-up?’ It’s problematic … so I prefer to have my operation in the Netherlands.” (R08).*

Positive experiences with the healthcare services in Turkey confirmed their belief that this was the healthcare system they preferred. Moreover, the respondents were now positive about utilizing healthcare in Turkey in the future and recommending it to others.

## Discussion

Dutch citizens of Turkish origin utilized healthcare in Turkey on an opportunistic basis, motivated by their beliefs of what constitutes “good care”, perceived unmet needs for specialist care, and guided by their social network. The social network played a large role in tipping the scales when deciding whether to utilize such care. Respondents perceived Dutch GPs as passive and as being reluctant to refer them to specialist care. However, these respondents also believed that GPs do not make the final decision about referring patients, since the Dutch healthcare system does not necessarily encourage referrals. Respondents perceived a need for specialist care and expected active interventions (i.e., diagnostic work-ups and treatment that went beyond providing only painkillers). Such perceived unmet needs greatly increased the likelihood of utilizing healthcare services in Turkey if the opportunity arose.

### Our findings in comparison with the existing literature

Our study provides possible explanations for the relatively high percentage of healthcare utilization among Dutch residents of Turkish origin in Turkey in our previous study, why they were dissatisfied with the care they received in the Netherlands, and why they had a perceived need for a second opinion [9].

Four other qualitative studies found similar patient- and system-related factors [4, 5, 8, 28]. A study on South Korean migrants in New Zealand found that the main motives were prior HCUCO, lingo-cultural barriers within the healthcare system, a consumerist view of healthcare (i.e. a commodity that should be available on demand), and having transnational lives (i.e., visiting family and friends in, and feeling connected with South Korea) [5]. These findings are in line with South Korean migrants living in Canada who also perceived sociocultural barriers in accessing healthcare in Canada, which led to a greater likelihood of HCUCO [8]. All perceived barriers in Canada and New Zealand were linked to either GP care or to differences in medical cultures [5, 8]. Mexican migrants living in the United States mentioned the same motives, after the main motive of cost of healthcare. They utilized healthcare in Mexico because of the personal attention provided, and the rapidity and efficacy of services [4]. These findings are further supported by a Danish study in which themes for healthcare services use in the region of origin were related to availability of access to medical specialist care, familiarity with the healthcare system in the country of origin, perception of good clinical practice, and the perceived need for care [28].

All findings except language barriers are in line with our data. The respondents in our study felt that language barriers were not important factors in healthcare utilization. However, they did admit that it was for others easier to communicate in one’s own language. In addition, they considered it to be a main reason for utilising healthcare in the country of origin for those experiencing communication problems. When respondents were asked if this also applied to their own situation, all of them denied having difficulty explaining their complaints to Dutch physicians. We believed that not being proficient in Dutch was seen as shameful and difficult for participants to admit. However, previous research has shown that language barriers always play a role and were often underestimated by both patients and healthcare providers, stressing the importance of overcoming language barriers [29–32].

Our data and the abovementioned qualitative studies suggest that not being able to access specialist care without a referral from the GP and differences in medical culture that lead to a mismatch in expectations, health beliefs, and perceived high need for care seem to be universal motives for migrants to utilize healthcare in their country of origin.

The data also suggest the possibility of certain hazards in continuity of care, namely, the consequences of cross-border care in terms of follow-up care, medication prescription, and transfer of medical information. Currently, follow-up care is arranged solely at the patient’s initiative, and prescribing of medication is not regulated or checked for duplications and missing prescriptions. In addition, no medical information is transferred, which leads to gaps in the patient’s medical history that can be vital in emergency situations (e.g., acute complications of an unknown treatment).

To address these hazards, we propose several implications for GP practice. GPs need to acknowledge that citizens of migrant origin are utilizing cross-border care, explore the expectations of and needs for care among these citizens, and seek common ground to enhance the patient-provider relationship [33]. Furthermore, GPs should provide the opportunity to discuss cross-border care, take a neutral attitude, provide patients with knowledge on the possible consequences of HCUCO, and take the initiative to maintain continuity of care. GPs can take the first step in continuity of care by providing patients with a brief letter containing their medical history, along with an up-to-date medication list, and instructing them to ask for a discharge letter after visiting an outpatient clinic in the country of origin.

Problems that occur because of a mismatch in needs could be addressed by educating patients and their social networks on how the healthcare system in the country of residence is organized, how common health problems are dealt with, and why a referral system to specialist care is in place. This kind of education should be available to both established and new citizens of migrant origin.

To ensure continuity of care for cross-border patients, future research should focus on how to formally organize cross-border communication between healthcare providers.

### Strengths and limitations

The strength of our study lies in the data collection, which was done in Turkish hospitals by a researcher of the same ethnic origin in collaboration with the public health department of a Turkish university. Because the researcher who collected the data (the first author) was bilingual, nothing was lost in translation. Other important strengths were the fact that we interviewed respondents during the healthcare utilization process, which meant there was no recall bias, and that the first author translated the interviews from Turkish to English, which made it possible for the other authors to review the coding and theme analyses of transcripts. All interviews, transcripts, and field notes are digitally available and securely stored at the Department of Public Health at the Academic Medical Center, University of Amsterdam according to the research guidelines of the department.

Our study’s main limitation is that the period in which the interviews took place was limited to two months (i.e., the summer vacation period of Dutch citizens). In addition, the first author sometimes missed a potential respondent due to the inability to be in two places at once.

## Conclusion

Our qualitative study shows that, among citizens of migrant origin, perceived unmet healthcare needs may arise due to differences between health and healthcare services beliefs among citizens of migrant origin and previous health experiences with their GP in the country of residence. This, in turn, leads to a perception of suboptimal care among these citizens, which motivates them to seek a referral for specialist care, and, if denied, to seek treatment in their country of origin. Quite often, the social network enables and facilitates the seeking of such care in the country of origin.

## Additional file

Additional file 1: Interview guide, topic list for interviewing Dutch residents of Turkish origin utilising healthcare in Turkey. (DOCX 80 kb)

## Abbreviations

EEA: European Economic Area
EHIC: European Health Insurance Card
EU: European Union
GP: General Practioner
HCUCO: Healthcare utilization in the country of origin
HTP: Health Transformation Program
