# Supplementary material for: Dutch citizens of Turkish origin who utilize healthcare services in Turkey: a qualitative study on motives and contextual factors
Source: BMC Health Serv Res. 2018 Apr 17;18:289. doi: 10.1186/s12913-018-3026-9 (PMC5905158; doi:10.1186/s12913-018-3026-9)
Supplement: Supplementary file 1 — Interview guide, topic list for interviewing Dutch residents of Turkish origin utilising healthcare in Turkey. (DOCX 80 kb) [file 12913_2018_3026_MOESM1_ESM.docx]

| Topic List |  |  |  |  |
| --- | --- | --- | --- | --- |
| Themes | **Subthemes** | | **Hypotheses** | **Examples** |
| Pathway |  | |  |  |
|  | Route to healthcare provider | | Information gathering, choice, contact, insurance | Arranging outpatient clinic visit, deliberate choice of healthcare provider, contact with insurance for reimbursement, advice or avoiding of contact |
|  | Obstacles | | Reimbursement, travel, other | Costs not covered by insurance, out-of pocket travel expenses |
|  |  | |  |  |
| Reasons |  | |  |  |
|  | Push- and pull factors (divided into Dutch Healthcare system or Turkish Healthcare System) | | (Previous) experiences, promotion (e.g. advertisements, discount, free additional service for patient bonding), recommendation, perceived quality of care; and self-reported approachability, acceptability, availability and accommodation, affordability, appropriateness, costs, culture and social factors (perceived cultural distance to the healthcare system, health literacy), privacy and hospitality | Push factors: Satisfaction with Dutch GP or specialist, influence of caregivers, family, acquaintances, waiting time to treatment, ramifications of complaints, decision-making style, education level, mastery of Dutch, miscommunication, understanding medical information in the country of residence / origin  Pull factors: Satisfaction with Turkish healthcare provider, advertisements, reputation, ease of use, supplementary services provided by healthcare provider (i.e. local transportation, accommodation, handling insurance forms and services, etc.), healthcare system of reference, preference for the difference, in consultation, treatment, decision-making style, perceived need to visit a doctor, and access to specialist care, familiarity, insurance coverage of treatment (out-of-pocket costs), healing environment (private room, own nurse) |
|  | Facilitating factors | | Caregivers, family, acquaintances | Influence of family in Turkey/Netherlands, company during healthcare utilisation, role of caregivers, family, acquaintances (e.g. civil status) |
| Expectations |  | |  |  |
|  | Goal | | Assurance, confirmation | Diagnosis, treatment, second opinion |
|  | Choice of healthcare provider | | Recommendation, promotion, quality of care | Influence of caregivers, family, acquaintances, advertisements, expertise, reputation |
| Experiences |  | |  |  |
|  | Achievement of goal | |  |  |
|  | Safety | | Informed decision making, complications of treatment | Aware of any risks associated with the treatment, presented a choice in treatment, unexpected adverse events |
| Consequences |  | |  |  |
|  | View on healthcare consumption in the country of origin | | Experienced health changes, future health problems, recommendation | Improvement of illness, favourable for utilising healthcare in the country origin, suggestion to caregivers, family, acquaintances. |
|  | After return | | Social network, healthcare providers | Reaction of caregivers, family, acquaintances, Dutch GP, Specialist of insurer |
|  | Continuity of care | | Arrangement, requirements, responsibility | Visits to outpatient clinic in the country of origin, healthcare provider in the country of residence |
|  | Follow-up | | Availability, organisation, transfer of medical records to the country of residence | Healthcare provider in the country of origin or country of residence |
|  | Unexpected effects | | General |  |

Topic list for interviewing Dutch residents of Turkish origin utilising healthcare in Turkey
